# Molecular Evidence of Increased Resistance to Anti-Folate Drugs in *Plasmodium falciparum* in North-East India: A Signal for Potential Failure of Artemisinin Plus Sulphadoxine-Pyrimethamine Combination Therapy

**DOI:** 10.1371/journal.pone.0105562

**Published:** 2014-09-03

**Authors:** Pradyumna Kishore Mohapatra, Devojit Kumar Sarma, Anil Prakash, Khukumoni Bora, Md. Atique Ahmed, Bibhas Sarma, Basanta Kumar Goswami, Dibya Ranjan Bhattacharyya, Jagadish Mahanta

**Affiliations:** 1 Regional Medical Research Centre, NE Region (ICMR), Dibrugarh, Assam, India; 2 National Institute for Research in Environmental Health (ICMR), Kamla Nehru Hospital Building, Gandhi Medical College Campus, Bhopal, Madhya Pradesh, India; Centro de Pesquisa Rene Rachou/Fundação Oswaldo Cruz (Fiocruz-Minas), Brazil

## Abstract

North-east India, being a corridor to South-east Asia, is believed to play an important role in transmitting drug resistant *Plasmodium falciparum* malaria to India and South Asia. North-east India was the first place in India to record the emergence of drug resistance to chloroquine as well as sulphadoxine/pyrimethamine. Presently chloroquine resistance is widespread all over the North-east India and resistance to other anti-malarials is increasing. In this study both *in vivo* therapeutic efficacy and molecular assays were used to screen the spectrum of drug resistance to chloroquine and sulphadoxine/pyrimethamine in the circulating *P. falciparum* strains. A total of 220 *P. falciparum* positives subjects were enrolled in the study for therapeutic assessment of chloroquine and sulphadoxine/pyrimethamine and assessment of point mutations conferring resistances to these drugs were carried out by genotyping the isolates following standard methods. Overall clinical failures in sulphadoxine/pyrimethamine and chloroquine were found 12.6 and 69.5% respectively, while overall treatment failures recorded were 13.7 and 81.5% in the two arms. Nearly all (99.0%) the isolates had mutant *pfcrt* genotype (76T), while 68% had mutant *pfmdr*-1 genotype (86Y). Mutation in *dhps* 437 codon was the most prevalent one while *dhfr* codon 108 showed 100% mutation. A total of 23 unique haplotypes at the *dhps* locus and 7 at *dhfr* locus were found while *dhps*-*dhfr* combined loci revealed 49 unique haplotypes. Prevalence of double, triple and quadruple mutations were common while 1 haplotype was found with all five mutated codons (**F/AGEGS/T**) at *dhps* locus. Detection of quadruple mutants (51I/59R/108N/164L) in the present study, earlier recorded from Car Nicobar Island, India only, indicates the presence of high levels of resistance to sulphadoxine/pyrimethamine in north-east India. Associations between resistant haplotypes and the clinical outcomes and emerging resistance in sulphadoxine/pyrimethamine in relation to the efficacy of the currently used artemisinin combination therapy are discussed.

## Introduction

Control of *Plasmodium falciparum* (Pf) malaria in the north-east India, reporting 0.15–0.2 million cases of malaria and over 100 deaths annually [1] is becoming challenging due to the widespread resistance to chloroquine (CQ) and increasing therapeutic failure to sulphadoxine/pyrimethamine (SP) [2]. Chloroquine resistance in Pf first appeared more or less simultaneously in Southeast Asia (Thai-Cambodian border) and South America (Colombia) in the late 1950s [3], [4]. In India, resistance to chloroquine in Pf was first detected in 1973 in Diphu area of Karbi-Anglong district of Assam state, north-east India [5] which gradually spread towards the east, west and south, covering almost entire country [6]. While resistance to SP was first reported again from Thai-Cambodian border in 1960s [7], initial reports of SP resistance in India, emerged in 1979, again in Karbi-Anglong district, Assam [8] and later documented in 1992 in Changlang district of Arunachal Pradesh, north-east India [9] indicating epidemiological importance of this region in spreading the drug resistant strains. Recently, treatment failure to CQ, SP and quinine, administered sequentially, was noted in 6% of Pf cases, indicating the presence of multidrug resistant Pf strains [10]. It has been observed that though the slide positivity rates (SPR) and slide falciparum rates (SFR) in north-east India have increased following emergence of drug resistance, the trend of progression of SFR over time has remained more or less linear, the case fatality rate (CFR) showed a steady increase (Figure S1 & Figure S2 as per north-east Indian states NVBDCP data).

Resistance to anti-folates (e.g. sulphadoxine/pyrimethamine) has been found associated with the point mutations at dihydrofolate reductase (*dhfr*) and dihydropteroate synthetase (*dhps*) genes and that of chloroquine with Pf chloroquine resistance transporter (*Pfcrt*) and Pf multidrug resistance (*Pfmdr1*) genes, [11], [12], [13]. Mutations at several amino acid positions of the *Pfcrt* gene from the chloroquine resistant parasite lines have been reported, of which mutation at 76 position from lysine to threonine (K76T) was consistently present among CQ-resistant *P. falciparum* isolates [14], making it a good marker to provide information on the Pf chloroquine resistance status. Additionally, point mutations at N86Y, Y184F, N1042D and D1246Y loci of *pfmdr*-1 gene are reported to reduce susceptibility to chloroquine [15], [16]. Pyrimethamine inhibits the parasite enzyme *Pfdhfr* involved in folate biosynthesis pathway [17], [18]. However, parasites with point mutations at codon position 51, 59, 108 and 164 of *dhfr* gene require higher amount of pyrimethamine to inhibit its activity and subsequently become resistant to pyrimethamine. The first mutation was found to be in position 108 (S108N). The drug sensitivity is reduced further if this mutation occurs along with mutations at N51I (Asn-Ile), C59R (Cys-Arg) or I164L (Ile-Leu). It was noted that the double mutation in *dhfr* gene (N51I+S108N or C59R+S108N) causes intermediate resistance while the triple mutation of the codons S108N+N51I+C59R confers high resistance [19]. However, an additional mutation at codon I164L leading to a quadruple mutation results in highest level of resistance to SP as well as to chloroproguanil dapsone and artesunate dapsone proguanil [20]. Sulphadoxine competes with the substrate that binds to the parasite enzyme Pfdhps. Mutations at several amino acid positions of this enzyme have shown reduction in its binding capacity to the drug. Mutations in *pfdhps* gene have been reported at five different amino acid positions (436, 437, 540, 580 and 613). In *dhps* mutation first starts at codon S436F/A (Ser-Ala/Phe) or at A437G (Ala-Gly) followed by mutations at other positions *viz.*, K540E (Lys-Glu), A581G (Ala-Gly) and A613S/T (Ala-Ser/Thr) conferring more resistance [1], [21], [22], [23], [24], [25], [26]. The presence of mutations at codons 437 and 540 of *pfdhps* together with the triple mutation of *pfdhfr* [quintuple mutation] is a significant predictor of SP treatment failure [27].

CQ was the drug of choice in north-east India, except in some select areas where SP was used as the first line of treatment till 2008. Thorough assessment of the extent of anti-malarial drug resistance and therapeutic failure is required before taking a call on making any changes in the drug policy, particularly in highly endemic areas such as north-east India. In view of this, the present study was conducted during 2006–2007 in two districts of Assam state in north-east India covering two sites located at considerable distances to determine the therapeutic efficacy of CQ and SP against uncomplicated falciparum malaria. Further, the frequency of mutations associated with CQ and SP resistance were determined in circulating Pf strains to correlate their association with therapeutic efficacy.

## Materials and Methods

### Study sites and study design

**Figure 1:**
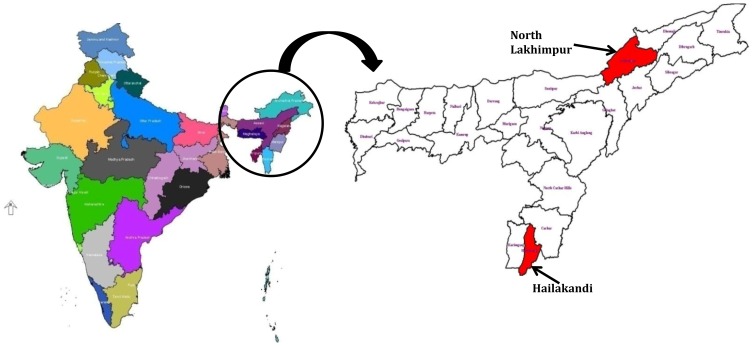
Map of the studied areas of north east India.

The study was carried out in two malaria endemic districts of north-east Indian state of Assam *viz*., North Lakhimpur and Hailakandi (Figure 1). Fever cases attending the Primary Health Centre of these two study sites were screened for malaria infection using conventional microscopy by examining both thick and thin blood smears stained with 3% Giemsa. Uncomplicated falciparum malaria cases meeting the following inclusion criteria were included in the study (1) >6 months of age (2) initial parasite count within 1000–1,00,000 parasites/µl on Day 0 (3) not suffering from severe malnutrition (4) mono infection with *P. falciparum* (5) no signs of any other illness and other causes of febrile disease (6) axillary temperature ≥37.5°C (7) written informed consent for willingness to participate in the study (8) no history of allergic reactions to sulfa-containing drugs and (9) able to return to clinic for follow-up visits.

A total of 220 Pf positives subjects fulfilling above criteria were enrolled in the study at PHC Kathlicherra in Hailakandi district (n = 120) and PHC Nowboicha in North Lakhimpur district (n = 100) and the therapeutic assessment of CQ and SP was carried out. Both drugs were supplied by the Directorate of National Vector Borne Diseases Control Programme, Government of India. CQ was administered orally at 25 mg/kg body weight in three divided doses (10 mg/kg on Day 0, 10 mg/kg on Day 1 and 5 mg/kg on Day 2). SP was given orally as a single dose at 25 mg sulphadoxine and 1.25 mg pyrimethamine/kg body weight. Antimalarials were administered under direct supervision of the investigators and patients were followed-up for a period of 28 days for CQ and 42 days for SP. Patients showing failure were treated with parental quinine in 5% dextrose until they were able to take the drug orally. All patients were given 0.75 mg primaquine/kg body weight as a single dose, for radical cure, at the end of the study.

### Clinical and Parasitological evaluation

The standard WHO protocol [28] for classification of response to anti-malarial treatment in low to moderate transmission areas was adopted. Blood smears were taken for parasite density count on day 0, 1, 2, 3, 7, 14, 21, 28 and 42 of recruitment along with the recording of axillary temperature and clinical assessment of the enrolled subjects. In addition, blood samples were collected from the willing subjects on filter paper on day 0 for molecular studies. The clinical response was classified as (i) early treatment failure (ETF) (ii) late clinical failure (LCF) (iii) late parasitological failure (LPF) and (iv) adequate clinical and parasitological response (ACPR).

### Sample processing and DNA Isolation

Approximately 50 µl of blood from each fever case at the time of initial recruitment was spotted on Whatman no. 1filter paper, desiccated and transferred to the laboratory. Parasite DNA was extracted from 4–5 punches (3 mm diameter) cut from dried blood spot from each filter paper using QIAamp DNA Blood Mini Kit (Cat. 51106, QIAGEN, USA) following manufacturer's instructions. The parasite DNA was eluted in 50 µl volume and stored at 4°C for immediate use. The parasite species was determined using primers and method described earlier [29].

### Parasite genotyping

**Figure 2:**
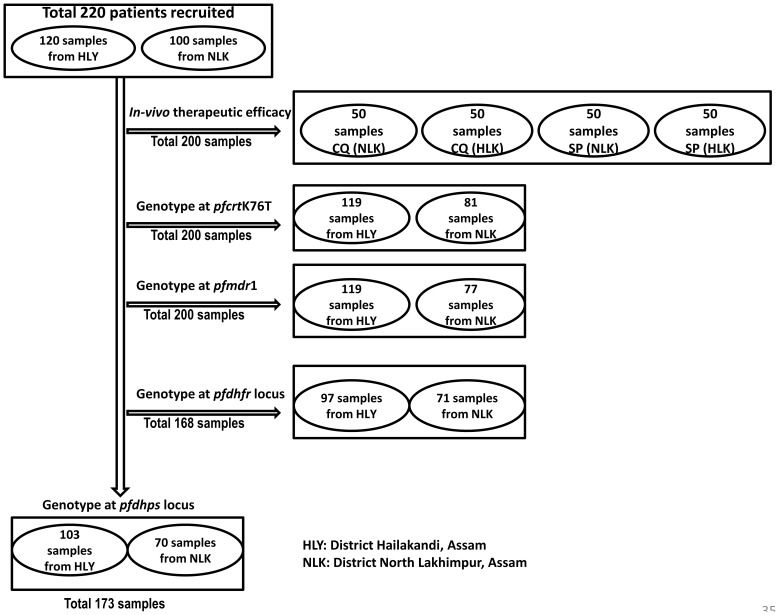
Sample size, site and genotyped codon wise.

*P. falciparum* mutations associated with CQ and SP resistance were typed by nested PCR-RFLP method as described and available on the web (http://medschool.umaryland.edu/CVD/plowe.html). In this study, the following codons and polymorphisms were analyzed: *pfmdr* 86, *pfcrt* 76; *pfdhfr* (16, 50, 51, 59, 108, 164) and *pfdhps* (436, 437, 540, 581, 613). All PCR amplifications were performed with MJ Mini thermal cycler (Bio-Red) in a reaction volume of 25 µl with Promega master mix. 5 µl of extracted DNA was used as a template in 1st round PCR while 1–2 µl of primary PCR product was used as a template for nested PCR. 5–8 µl of 2nd round PCR product was digested with 1 unit of respective restriction enzyme (NEB) in a 20 µl reaction volume using NEB buffer. Amplicons and fragments were separated on 2% or 3% agarose gels, stained with ethidium bromide and visualized under UV. The detailed description of sample size used for different aspects are outlined in Figure 2.

### Data analyses

The data was double entered in SPSS V13.0. All statistical analyses were carried out using SPSS V13.0 and XLSTAT V-2012.5.02.

Mutations were determined on the basis of the presence or absence of the expected bands on ethidium bromide stained agarose gel analysis from PCR amplicon after restriction digestions. Site specific distribution of allele was expressed in percentage. *dhps* and *dhfr* haplotypes were counted separately and in combination and their prevalence recorded. Associations between *dhfr/dhps* alleles and SP therapeutic failure and *pfcrt* alleles and CQ therapeutic failure were assessed using an adjusted Odds ratios (OR) and the 95% confidence intervals. Appropriate statistical tests were carried out.

### Ethical consideration

The study was approved by the Scientific Advisory Committee and Human Ethical Committee of Regional Medical Research Centre, Dibrugarh, Assam (India). All participants provided informed written consent. However, in case of a minor or children enrolled in the study, informed written consent was taken either from their parents or from their legal guardians, if parents were not available. As proposed by the Institutional Ethics Committee, Regional Medical Research Centre, Dibrugarh, Assam, all participants were informed about the objectives and intended use of the findings prior to their enrollment in the study as well as their right to withdraw from the study at any time without notification. The informed consent was translated into local language.

## Results

### Therapeutic efficacy of CQ and SP

A total of 200, among the 220 enrolled subjects with acute uncomplicated malaria, from the two sites were included in the study (50 for CQ and 50 for SP from each site) and treated with SP and CQ. Patients' characteristics, demographic data and treatment outcomes are shown in Table 1. Of these, 95 subjects each successfully completed the 28 days follow up for CQ and 42 days follow up for SP. The therapeutic efficacy study at the two sites revealed 86.3% and 19% ACPR response with SP and CQ respectively. Overall clinical failures in SP and CQ arms were 12.6 and 69.5% respectively, while overall treatment failures were 13.7 and 81.5% in the two arms. Hailakandi recorded the higher CQ failure while both the sites had almost similar rate of SP failure (Table 1).

**Table 1:** Demographic and clinical characteristics of patients and their responses to treatment with SP and CQ.

		Treatment with SP	Treatment with CQ
Sex	Male	66	66
	Female	34	34
	Male: Female	1.94	1.94
Age (years)	Mean ± S.D.	20.69±13.0	21.74±13.84
	Range	2–64	2–65
	n (<15 years)	34	33
	n (15–30 years)	45	44
	n (>30 years)	21	23
Parasites count (µL^−1^)	Geometric mean	19589.06	7678.79
	Range	3160–127720	1000–97866
	Proportion of patients with <50000	87	95
	Proportion of patients with >50000	13	5

Treatment outcome at day 28		Hailakandi	North Lakhimpur	Total	Hailakandi	North Lakhimpur	Total
	No. of included patients	50	50	100	50	50	100
	Loss	2	3	5	2	3	5
	Evaluable patients	48	47	95	48	47	95
	ACPR, n (%)	41(85.42)	41(87.23)	82(86.32)	5(10.42)	13(27.65)	18(18.95)
	ETF, n (%)	2(4.16)	3(6.38)	5(5.26)	28 (58.33)	21 (44.68)	49(51.58)
	LCF, n (%)	4(8.33)	3(6.38)	7(7.37)	8 (16.67)	9 (19.15)	17(17.89)
	**Total CF, n (%)**	**6(12.5)**	**6(12.77)**	**12(12.63)**	**36(75.0)**	**30(63.83)**	**66(69.47)**
	LPF, n (%)	1 (2.08)	0 (0.0)	1 (1.05)	7(14.58)	4(8.51)	11(11.58)
	**Overall TF, n (%)**	**7(14.58)**	**6(12.76)**	**13(13.68)**	**43(89.58)**	**34(72.34)**	**77(81.05)**

ACPR = Adequate Clinical and Parasitological Response; TF = Treatment failure, ETF = Early Treatment Failure, LCF = Late Clinical Failure; LPF = Late Parasitological Failure; CF = Clinical failure, TF = Treatment failure.

### Prevalence of *pfcrt* and *pfmdr*1 mutations

**Figure 3:**
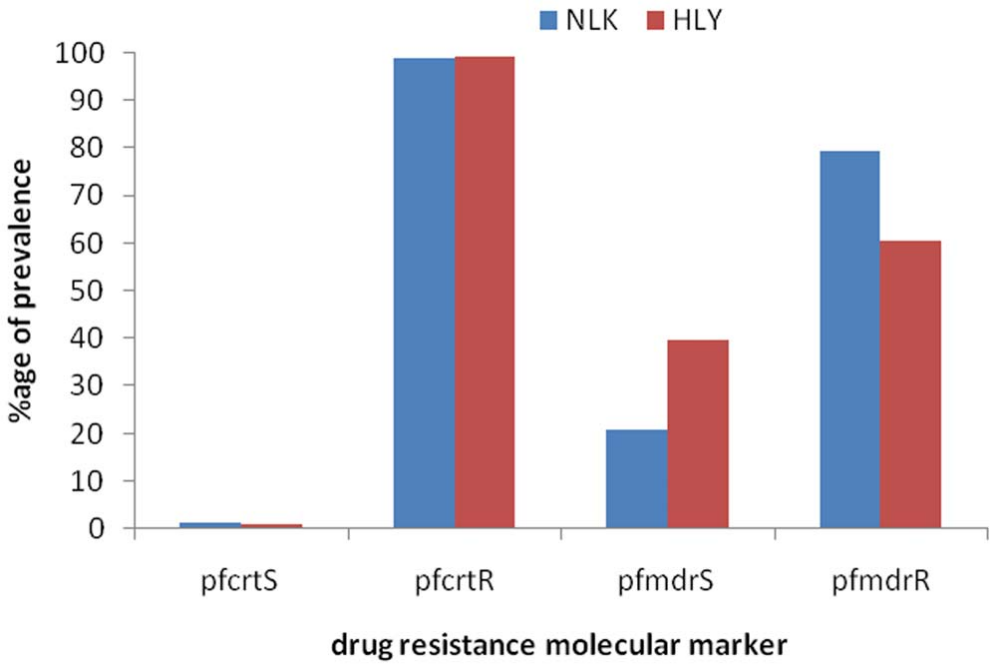
Prevalence of point mutations at *pfcrt* and *pfmdr1* locus in the two studied sites.

The status of *pfcrt* K76T and *pfmdr*-1 N86Y mutations was determined in 200 (119 from Hailakandi, 81 from North Lakhimpur) and 196 (Hailakandi = 119, North Lakhimpur = 77) isolates from the two locations. Based on *pfcrt* locus almost all isolates were found resistant to CQ at both the sites [North Lakhimpur 80/81 (98.8%), Hailakandi 118/119 (99.2%)], while at the *pfmdr*-1 locus 80% (61/77) isolates from North Lakimpur and 61% (72/119) isolates from Hailakandi were found resistant. Overall, 99% isolates had mutant *pfcrt* genotype (76T), while 68% had mutant *pfmdr*-1 genotype (86Y) locus (Figure 3).

### Prevalence of *dhps* and *dhfr* mutations

The presence of five *dhps* mutations (S436F/A, A437G, K540E, A581G, and A613S/T) and six *dhfr* mutations (A16V, C50R N51I, C59R, S108N and I164L) known to be involved in resistance to SP were evaluated (Table 2). Mutation analysis was successful in 173 subjects for *dhps* codons and in 168 patients for *dhfr* codons.

**Table 2:** Prevalence of *dhps* and *dhfr* mutations in isolates of *Plasmodium falciparum*.

Locus	Codons	Status	Total (n = 173)	Location	
				Hailakandi (n = 103)	North Lakhimpur (n = 70)
*dhps*	436	Wild (S)	130 (75.1)	70 (68.0)	60 (85.7)
		Mutant (F/A)	43 (24.9)	33 (32.0)	10 (14.3)
	437	Wild (A)	70 (40.5)	35 (34.0)	35 (50.0)
		Mutant (G)	103 (59.5)	68 (66.0)	35 (50.0)
	540	Wild (K)	106 (61.3)	48 (46.6)	58 (82.9)
		Mutant (E)	67 (38.7)	55 (53.4)	12 (17.1)
	581	Wild (A)	136 (78.6)	78 (75.7)	58 (82.9)
		Mutant (G)	37 (21.4)	25 (24.3)	12 (17.1)
	613	Wild (A)	134 (77.5)	75 (72.8)	59 (84.3)
		Mutant (S/T)	39 (22.5)	28 (27.2)	11 (15.7)

Locus	Codons	Status	Total (n = 168)	Location	
				Hailakandi (n = 97)	North Lakhimpur (n = 71)
*dhfr*	16	Wild (A)	166 (98.8)	95 (97.9)	71 (100.0)
		Mutant (V)	2 (1.2)	2 (2.1)	0
	50	Wild (C)	168 (100.0)	97 (100.0)	71 (100.0)
		Mutant (R)	0	0	0
	51	Wild (N)	134 (79.8)	77 (79.4)	57 (80.3)
		Mutant (I)	34 (20.2)	20 (20.6)	14 (19.7)
	59	Wild (C)	5 (3.0)	2 (2.1)	3 (4.2)
		Mutant (R)	163 (97.0)	95 (97.9)	68 (95.8)
	108	Wild (S)	0	0	0
		Mutant (N)	168 (100.0)	97 (100.0)	71 (100.0)
	164	Wild (I)	144 (85.7)	89 (91.8)	55 (77.5)
		Mutant (L)	24 (14.3)	8 (8.2)	16 (12.5)

Values within bracket are the percentage of occurrences.

**Figure 4:**
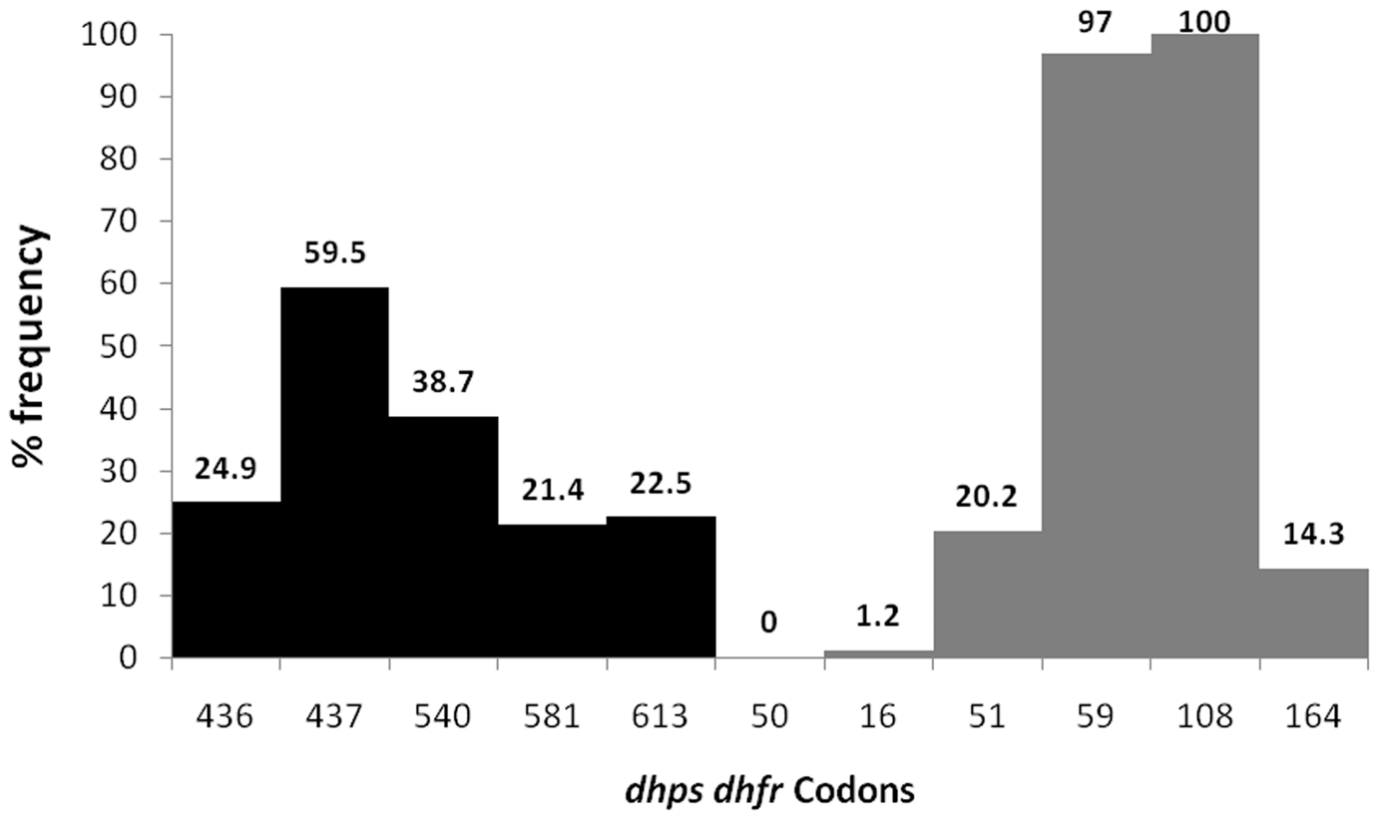
Prevalence of *dhps* and *dhfr* point mutations in the study.

Mutant codons were quite common at both the loci and study sites. Among all mutations studied, mutation in *dhps* 437 codon was the most prevalent, while mutation in *dhps* 581 codon was the least prevalent. Mutant alleles of *dhps* were more common in Hailakandi. The mutant *dhfr* 50 allele was completely absent while *dhfr* codon 108 showed 100% mutation (Table 2, Figure 4).

### Distributions of *dhps* and *dhfr* haplotypes

The allelic pattern or haplotypes were constructed for *dhps* and *dhfr* loci individually as well as in combination. A total of 23 unique haplotypes at the *dhps* locus (Table 3) and 7 haplotypes at *dhfr* locus (Table 4) were found. In case of *dhps*, 40 isolates (23.1% of isolates) had all five sensitive codons (SAKAA). On the other hand 5 haplotypes (22.5%) with single mutation (SAKA**S/T**, SAK**G**A, SA**E**AA, S**G**KAA, **F/A**AKAA), 7 haplotypes (25.4%) with double mutations (SAK**GS/T**, SA**E**A**S/T**, S**G**KA**S/T**, S**G**K**G**A, S**GE**AA, **F/A**A**E**AA, **F/AG**KAA), 6 haplotypes (22.5%) with triple mutations (S**G**K**GS/T**, S**GE**A**S/T**, S**GEG**A, **F/A**A**E**A**S/T**, **F/AG**KAA, **F/AGE**AA), 3 haplotypes (5.8%) with quadruple mutations (S**GEGS/T**, **F/AGE**A**S/T**, **F/AGEG**A) and 1 haplotype (0.6%) with all five mutated codons (**F/AGEGS/T**) were found. Higher prevalence of resistant allele at codon 437 (14 haplotypes), codon 540 (12 haplotypes) and codon 613 (11 haplotypes) was noted suggesting that mutations first started at 437 codon position of *dhps* locus in NE India and subsequently associated with other mutations to confer resistance (Table 3).

**Table 3:** The frequency distribution of SNPs combination for *dhps* alleles.

*dhps* haplotypes	Total (n = 173)	Location	
		Hailakandi (103)	North Lakhimpur (70)
SAKAA	40 (23.1)	15 (14.6)	25 (35.7)
SAKA**S/T**	7 (4.0)	3 (2.9)	4 (5.7)
SAK**G**A	7 (4.0)	6 (5.8)	1 (1.4)
SAK**GS/T**	1 (0.6)	1 (1.0)	0
SA**E**AA	6 (3.5)	4 (3.9)	2 (2.9)
SA**E**A**S/T**	2 (1.2)	1 (1.0)	1 (1.4)
S**G**KAA	17 (9.8)	5 (4.9)	12 (17.1)
S**G**KA**S/T**	3 (1.7)	2 (1.9)	1 (1.4)
S**G**K**G**A	13 (7.5)	6 (5.8)	7 (10.0)
S**G**K**GS/T**	9 (5.2)	6 (5.8)	3 (4.3)
S**GE**AA	16 (9.2)	13 (12.6)	3 (4.3)
S**GE**A**S/T**	4 (2.3)	4 (3.9)	0
S**GEG**A	3 (1.7)	3 (2.9)	0
S**GEGS/T**	2 (1.2)	1 (1.0)	1 (1.4)
**F/A**AKAA	2 (1.2)	0	2 (2.9)
**F/A**A**E**AA	4 (2.3)	4 (3.9)	0
**F/A**A**E**A**S/T**	1 (0.6)	1 (1.0)	0
**F/AG**KAA	5 (2.9)	2 (1.9)	3 (4.3)
**F/AG**KA**S/T**	2 (1.2)	2 (1.9)	0
**F/AGE**AA	20 (11.6)	16 (15.5)	4 (5.7)
**F/AGE**A**S/T**	7 (4.0)	6 (5.8)	1 (1.4)
**F/AGEG**A	1 (0.6)	1 (1.0)	0
**F/AGEGS/T**	1 (0.6)	1 (1.0)	0

Allelic combinations are in order of S436F/A, A437G, K540E, A581G, and A613S/T, where bold and underlined alleles denotes mutations. Values within bracket are the percentage of occurrences.

**Table 4:** The frequency distribution of SNPs combination of *dhfr* alleles.

*dhfr* haplotypes	Total (n = 168)	Location	
		Hailakandi (97)	North Lakhimpur (71)
ACNC**N**I	3 (1.8)	2 (2.1)	1 (1.4)
ACN**RN**I	108 (64.3)	67 (69.1)	41 (57.7)
**V**CN**RN**I	2 (1.2)	2 (2.1)	0
AC**IRN**I	31 (18.5)	18 (18.6)	13 (18.3)
ACNC**NL**	2 (1.2)	0	2 (2.8)
ACN**RNL**	19 (11.3)	6 (6.2)	13 (18.3)
AC**IRNL**	3 (1.8)	2 (2.1)	1 (1.4)

Allelic combinations are in order of A16V, C50R N51I, C59R, S108N and I164L where bold and underlined allele denotes mutations. Values within bracket are the percentage of occurrences.

At the *dhfr* locus, only 7 haplotypes were observed among 168 isolate (Table 4). No haplotype having all the 6 sensitive codons was observed. There was only 1 haplotype (1.8% of isolates) carrying single mutation (ACNC**N**I), 2 haplotypes (65.5%) with double mutations (ACN**RN**I, ACNC**NL**), 3 haplotypes (14.3%) with triple mutations (**V**CN**RN**I, AC**IRN**I and ACN**RNL**) and 1 haplotype (1.8%) with quadruple mutation (AC**IRNL**). The prevalence of haplotype ACN**RN**I containing mutations at codons 59 and 108 had the highest frequency (64.3%).

**Figure 5:**
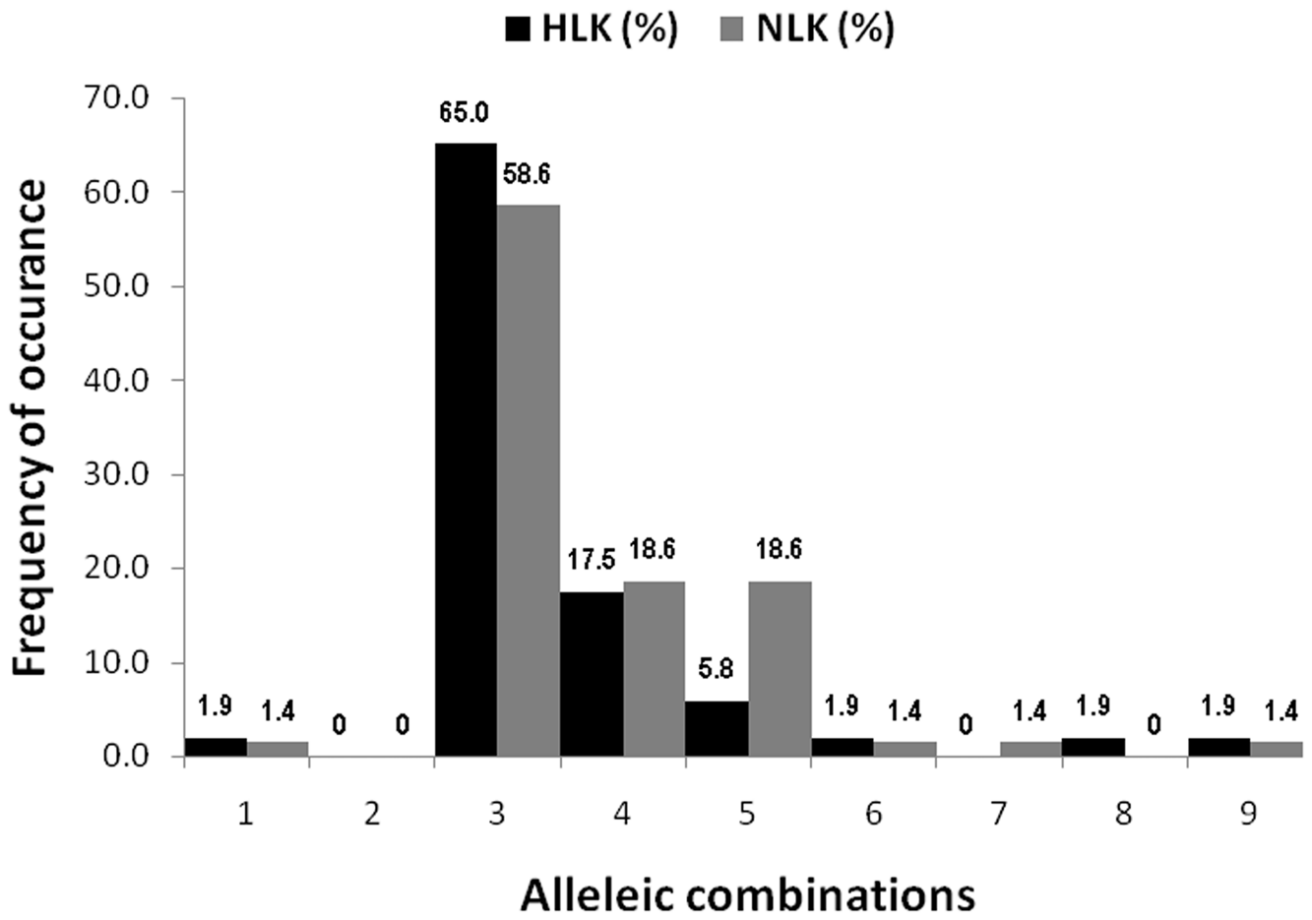
Frequency of allelic combinations mostly conferring resistance to SP.

Construction of *dhps*-*dhfr* combined haplotypes among the 154 isolates, where PCR-RFLP results for all codons were available, revealed 49 unique haplotypes, of which SAKAA-ACN**RN**I (13.6%), S**G**KAA-ACN**RN**I (8.4%) and **F/AGE**AA-ACN**RN**I (7.1%) haplotypes had the higher frequencies (Table 5). Assessment of *dhps*/*dhfr* combined haplotypes with single or multiple mutations *viz.*, S108N, N51I+S108N, C59R+S108N, N51I+C59R+S108N, C59R+S108N+I164L, N51I+C59R+S108N+I164L, N51I+C59R+S108N/A437G, N51I+C59R+S108N/K540E and N51I+C59R+S108N/A437G+K540E, considered as predictive for SP resistance or failure [26], revealed that C59R+S108N (n = 108; Hailakandi 65%, North Lakhimpur 59%), N51I+C59R+S108N (n = 31; Hailakandi 17.5%, North Lakhimpur 19%) and C59R+S108N+I164L (n  = 19; Hailakandi 6%, North Lakhimpur 19%) combinations were the most prevalent ones (Figure 5).

**Table 5:** The frequency distribution of SNPs combination of *dhps*/*dhfr* allele.

Sl. no.	*dhps+dhfr* combined	Frequency (n = 154)	Location		Sl. no.	*dhps+dhfr* combined	Frequency (154)	Location	
			Hailakandi (n = 88)	North Lakhimpur (n = 66)				Hailakandi (n = 88)	North Lakhimpur (n = 66)
1	**F/AGEGS/T**-ACN**RNL**	1 (0.6)	1 (1.1)	0	26	S**G**K**GS/T**-ACNC**N**I	1 (0.6)	0	1 (1.5)
2	**F/AGEGA**-ACN**RN**I	1 (0.6)	1 (1.1)	0	27	S**G**K**G**A-AC**IRN**I	3 (1.9)	3 (3.4)	0
3	**F/AGE**A**S/T**-AC**IRN**I	1 (0.6)	1 (1.1)	0	28	S**G**K**G**A-ACN**RNL**	1 (0.6)	0	1 (1.5)
4	**F/AGE**A**S/T**-ACN**RN**I	5 (3.2)	4 (4.5)	1 (1.5)	29	S**G**K**G**A-ACN**RN**I	9 (5.8)	3 (3.4)	6 (9.1)
5	**F/AGE**AA-AC**IRN**I	3 (1.9)	2 (2.3)	1 (1.5)	30	S**G**KA**S/T**-AC**IRNL**	1 (0.6)	1 (1.1)	0
6	**F/AGE**AA-ACN**RNL**	3 (1.9)	1 (1.1)	2 (3.0)	31	S**G**KA**S/T**-ACN**RN**I	3 (1.9)	2 (2.3)	1 (1.5)
7	**F/AGE**AA-ACN**RN**I	11 (7.1)	10 (11.4)	1 (1.5)	32	S**G**KAA-AC**IRN**I	1 (0.6)	0	1 (1.5)
8	**F/AGE**AA-ACNC**N**I	1 (0.6)	1 (1.1)	0	33	S**G**KAA-ACN**RNL**	1 (0.6)	0	1 (1.5)
9	**F/AG**KAA-AC**IRN**I	2 (1.3)	0	2 (3.0)	34	S**G**KAA-ACN**RN**I	13 (8.4)	4 (4.5)	9 (13.6)
10	**F/AG**KAA-ACN**RN**I	3 (1.9)	2 (2.3)	1 (1.5)	35	SA**E**AA-AC**IRN**I	2 (1.3)	2 (2.3)	0
11	**F/A**A**E**A**S/T**-ACN**RN**I	1 (0.6)	1 (1.1)	0	36	SA**E**AA-ACN**RNL**	1 (0.6)	0	1 (1.5)
12	**F/A**A**E**AA-ACN**RNL**	1 (0.6)	1 (1.1)	0	37	SA**E**AA-ACN**RN**I	2 (1.3)	2 (2.3)	0
13	**F/A**A**E**AA-ACN**RN**I	3 (1.9)	3 (3.4)	0	38	SA**E**AA-ACNC**NL**	1 (0.6)	0	1 (1.5)
14	**F/A**AKAA-ACN**RN**I	1 (0.6)	0	1 (1.5)	39	SAK**GS/T**-ACN**RN**I	1 (0.6)	1 (1.1)	0
15	S**GEGS/T**-AC**IRNL**	1 (0.6)	0	1 (1.5)	40	SAK**G**A-AC**IRN**I	1 (0.6)	1 (1.1)	0
16	S**GEGS/T**-ACN**RN**I	1 (0.6)	1 (1.1)	0	41	SAK**G**A-ACN**RN**I	6 (3.9)	5 (5.7)	1 (1.5)
17	S**GEG**A-AC**IRN**I	1 (0.6)	1 (1.1)	0	42	SAKA**S/T**-**V**CN**RN**I	1 (0.6)	1 (1.1)	0
18	S**GEG**A-ACN**RN**I	2 (1.3)	2 (2.3)	0	43	SAKA**S/T**-AC**IRN**I	3 (1.9)	2 (2.3)	1 (1.5)
19	S**GE**AA-AC**IRN**I	3 (1.9)	2 (2.3)	1 (1.5)	44	SAKA**S/T**-ACN**RNL**	1 (0.6)	0	1 (1.5)
20	S**GE**AA-ACN**RNL**	1 (0.6)	1 (1.1)	0	45	SAKA**S/T**-ACN**RN**I	2 (1.3)	0	2 (3.0)
21	S**GE**AA-ACN**RN**I	7 (4.5)	6 (6.8)	1 (1.5)	46	SAKAA-**V**CN**RN**I	1 (0.6)	1 (1.1)	0
22	S**GE**AA-ACNC**NL**	1 (0.6)	0	1 (1.5)	47	SAKAA-AC**IRN**I	9 (5.8)	4 (4.5)	5 (7.6)
23	S**GE**AA-ACNC**N**I	1 (0.6)	1 (1.1)	0	48	SAKAA-ACN**RNL**	7 (4.5)	1 (1.1)	6 (9.1)
24	S**G**K**GS/T**-AC**IRNL**	1 (0.6)	1 (1.1)	0	49	SAKAA-ACN**RN**I	21 (13.6)	8 (9.1)	13 (19.7)
25	S**G**K**GS/T**-ACN**RN**I	6 (3.9)	4 (4.5)	2 (3.0)					

Allelic combinations are in order of S436F/A, A437G, K540E, A581G, and A613S/T (*dhps*)-A16V, C50R N51I, C59R, S108N and I164L (*dhfr*) where bold alleles denotes mutations. Values within bracket are the percentage of occurrences.

### Association between *dhfr*-*dhps* point mutations and treatment failure with SP and CQ

**Figure 6:**
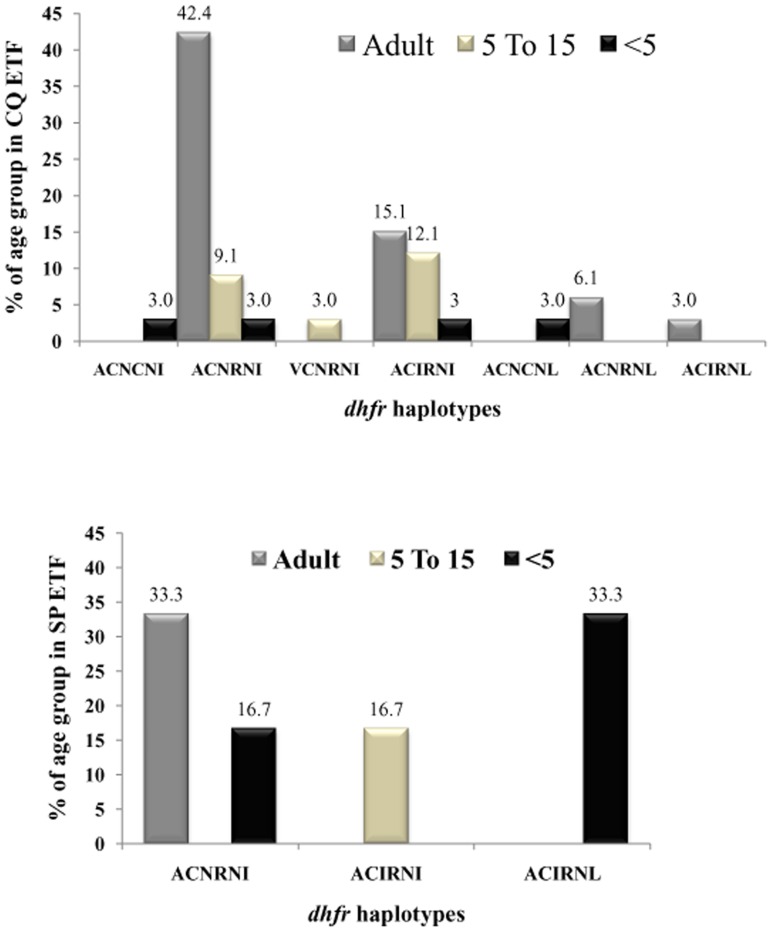
Age and codon wise association of CQ (above) and SP (below) ETF.

SP treatment failure was strongly associated with *dhps-dhfr* mutations and their combinations. It was found that 5 *dhps-dhfr* combined haplotypes were associated with 6 cases of early treatment failures of SP, while 25 *dhps-dhfr* combined haplotypes were associated with 33 cases of early treatment failure of CQ. Most of the treatment failure haplotypes were with mutations 59R+108N or 51I+59R+108N or 59R+108N+164L at the *dhfr* locus indicating their role in SP treatment failure in north-east India (Table 6). Among the CQ failure cases, 67% belonged to adult age group (>15 years), 24% to 5–15 years age and 9% were under 5 years of age. However, in case of SP treatment failures, 50% cases were below 5 years of age while >15 years and 5–15 years age groups represented 33% and 17% failure cases respectively. The *dhfr* double mutant ACN**RN**I (with mutations at 59 and 108 codon) with the highest frequency in the adult age group was associated with CQ treatment failure. However, in case of SP treatment failure, the same haplotype was found associated with treatment failure in adult cases (33.3%) as well as under 5 years of age (16.7%) while haplotype AC**IRNL** (with mutations at 51, 59, 108 and 164 codon) was solely associated with treatment failure cases in <5 years of age (Figure 6). Overall, the results were indicative of the presence of multi drug resistant strains of Pf in the study sites.

**Table 6:** *dhps/dhfr* haplotypes associated with CQ and SP early treatment failure (ETF).

*dhps/dhfr* haplotypes in CQ ETF			*dhps/dhfr* haplotypes in SP ETF		
Sl.No	Haplotypes	Frequency	Sl. No.	Haplotypes	Frequency
1	**F/AGE**A**S/T**-ACN**RN**I	2	1	S**G**K**G**A-ACN**RN**I	2
2	**F/AGE**AA-AC**IRN**I	1	2	S**G**KAA-ACN**RN**I	1
3	**F/AGE**AA-ACN**RN**I	2	3	SAK**G**A-AC**IRN**I	1
4	**F/AG**KAA-AC**IRN**I	1	4	S**G**K**GS/T**-AC**IRNL**	1
5	**F/AG**KAA-ACN**RN**I	1	5	S**G**KA**S/T**-AC**IRNL**	1
6	**F/A**A**E**A**S/T**-ACN**RN**I	1			
7	**F/A**A**E**AA-ACN**RNL**	1			
8	S**GEGS/T**-AC**IRNL**	1			
9	S**GE**AA-AC**IRN**I	1			
10	S**GE**AA-ACNC**NL**	1			
11	S**GE**AA-ACNC**N**I	1			
12	S**G**K**GS/T**-ACN**RN**I	1			
13	S**G**K**G**A-AC**IRN**I	1			
14	S**G**K**G**A-ACN**RN**I	2			
15	S**G**KA**S/T**-ACN**RN**I	1			
16	S**G**KAA-ACN**RNL**	1			
17	S**G**KAA-ACN**RN**I	2			
18	SA**E**AA-ACN**RN**I	1			
19	SAK**GS/T**-ACN**RN**I	1			
20	SAK**G**A-ACN**RN**I	1			
21	SAKA**S/T**-**V**CN**RN**I	1			
22	SAKA**S/T**-AC**IRN**I	1			
23	SAKA**S/T**-ACN**RN**I	1			
24	SAKAA-AC**IRN**I	4			
25	SAKAA-ACN**RN**I	2			

Allelic combinations are in order of S436F/A, A437G, K540E, A581G, and A613S/T (*dhps*)-A16V, C50R N51I, C59R, S108N and I164L (*dhfr*) where bold alleles denotes mutations.

## Discussion

Control of drug resistant *P. falciparum* malaria has become a challenging task globally. Except for artemisinin and its derivatives, anti-malarial resistance to *P. falciparum* has been reported *in vivo* for all drugs [30]. Chloroquine resistance to falciparum in South-east Asia originated from a single focus in Paillang region near Thai–Cambodia border during 1950s. Resistance to SP was detected in 1960s again from Thai-Cambodian border from where Pf strains resistant not only to CQ, but also to SP, mefloquine and quinine spread to surrounding countries [4]. This spread of anti-malarial resistance in South-east Asia apparently followed a pattern until 1978. Initially, it remained limited to tropical forested areas corresponding well with the geographical distribution of *Anopheles dirus* complex mosquitoes; thereafter, it moved out of the geographical limits of *An. dirus*. In India too, the same trend was witnessed where Pf strains resistant to CQ first appeared in 1973 [5] and to SP in 1987 [6] in *An. dirus* complex mosquito dominated areas of north-east India before moving to other parts of the country. Therefore, north-east India may be acting as the gateway to resistant falciparum strains in India. Increasing failure rates of anti-malarials in north-east India demands a close monitoring of the epidemiology and dynamics of the drug resistant Pf. Availability of easy and rapid molecular markers would greatly facilitate this process and allows overcoming difficulties in the use of traditional methods for characterizing anti-malarial drug resistance. Moreover, the molecular surveillance of drug resistance markers such as *pfcrt*, *pfmdr*-1, *dhfr, dhps* & *pfatp*-6 associated with anti-malarial treatment failure provides sufficient evidence to policy makers for a renewed thinking on the existing drug policy [31].

Genotyping of circulating Pf strains for CQ resistance revealed that almost all isolates were resistant at *pfcrt* locus, while 68% isolates showed resistance at *pfmdr*-1 locus. Simultaneous *in vivo* therapeutic study recorded 80% treatment failure to CQ. Age stratified analysis revealed that of all the CQ failure cases (77/95), 78% (25/32) belonged to <15 years of age including 100% cases (7/7) belonging to <5 years indicating the synergistic role of host immunity in the clearance of parasitaemia. Although treatment failure in the older age group (>15 years) was also pretty high (22%), it might partly be explained based on the fact that many adult subjects included in the study were not the permanent inhabitants of the study sites and had migrated from non/less endemic areas for temporary contractual works in search of livelihood and possibly had low immune status against malaria. Earlier studies in north-east India [2], [10] have also reported CQ resistance in more than 80% isolates of Pf indicating non-effectiveness of CQ over the region. Although *pfmdr*-1 has been thought to affect CQ resistance, however, no correlation between the prevalence of *pfcrt* mutation and *pfmdr*-1 point mutation was noted in the present study, which was also observed in earlier studies further strengthening the doubt over its involvement in CQ resistance [32], [33].

In case of *dhps*, though the prevalence of sensitive haplotype (SAKAA) was as high as 23%, double (25.4%), triple (22.5%) and quadruple (5.8%) mutant haplotypes were also present in good numbers along with a full box mutation (**F/AGEGS/T**) in one isolate from Hailakandi district. To the best of our knowledge this is the first study recording as many as 23 unique *dhps* haplotypes and a full box mutated haplotype anywhere in India. Earlier studies found 8 [34] and 15 [35] *dhps* haplotypes. Presence of the mutated codon 437G (frequency 60%) in most of our *dhps* haplotypes suggests that mutation in this locus started settling first at 437 position in north-east India, similar to the inference made by others [26].

At *dhfr* locus, codon 108 was found mutated in all the isolates while no mutation was seen in codon 50. Double, triple and quadruple mutations were noted resulting in 7 different but unique haplotypes of *dhfr*. Elsewhere in India, presence of double mutants (59R/108N) or single mutants (108N) were found to be common [22], [36]. Although the presence of triple mutants *viz*. 51I/59R/108N or 59R/108N/164L were observed from north-east India in ∼10% isolates [22], [37], no study has ever reported quadruple mutations (51I/59R/108N/164L) from this part of country. We found 3 isolates (∼2%) harboring quadruple mutants (51I/59R/108N/164L) in the present study (Table 4) which was earlier recorded only from Car Nicobar Island, India [37], [38]. We found the presence of double mutants of *dhfr* i.e., C59R/S108N or N51I/S108N associated with intermediate level of resistance to SP, while presence of triple mutant (N51I/C59R/S108N) was seen in 32% isolates of early treatment failure of SP in therapeutic assessment.

This study revealed high frequency of mutant codons at both *dhps* and *dhfr* loci that correlated well with the status of therapeutic response to SP in Pf malaria cases. Presence of haplotypes with mutations at codon position 437 and 540 in the *dhps* gene combined with triple and quadruple mutations in *dhfr* gene, seen in about 10% of isolates comprising of 8 haplotypes (Table 4), was predictive of early treatment failure of SP. Overall prevalence of mutated codons of *dhps* and *dhfr* in north-east India detected in our study matches well with the data available in the World Wide Antimalarial Resistance Network Molecular Surveyor (WWARN: http://www.wwarn.org/surveyor/) (Figure S3). From the distribution of the mutations, it can also be inferred that most of the mutations in *dhps* and *dhfr* loci are fixed in north-east India and is now spreading to other parts of India.

The present study provides important information not only on the resistance pattern of CQ and SP drugs to Pf in Assam state of north-east India (2006–2007) but also raises concern on the potential effectiveness of the current first line of the drug i.e. Artemisinin based combination therapy (ACT) along with highlighting the importance of constant molecular surveillance of drug resistance. ACT (Artesunate with SP) has been introduced in north-east India since 2008 as the first line of drug for treating uncomplicated *P. falciparum* cases. Present study indicates that although SP is therapeutically effective in treating uncomplicated Pf malaria cases in the study sites, yet fixation and prevalence of double, triple and even quadruple mutations at both the loci associated with its treatment failure was quite high.

Although we didn't explore *pfatp*6, the molecular marker for artemisinin resistance, in this study, as ACT was not in use during the study period, yet it can be speculated from the findings that due to wide spread occurrence of *dhfr* and *dhps* mutant codons in north- east India, the artesunate, that is combined with sulphadoxine-pyrimethamine in ACT, is probably acting as a monotherapy. Since artemisinin is a short acting drug with a rapid parasite reduction and a shorter half-life (in hours only), it is generally combined with a partner drug (such as SP) having longer half life with different mechanism of action. However, in areas of high transmission, like in north-east India, the partner drug may promote the proliferation of drug resistant parasites exposed to sub therapeutic levels of drug, which may result in decreased efficacy of ACT [39]. It is further speculated that the situation in north-east India might further worsen by changes in frequencies of mutant alleles with the passage of time due to the selective drug pressure which raises serious concern over using SP as a partner drug with artemisinin for the treatment of Pf cases. Therefore, current therapeutic efficacy studies of ACT in north-east India are urgently warranted. Although there is a paucity of data on the therapeutic efficacy of ACT from this part of India, yet a study on ACT involving 4 sites in the Myanmar bordering areas of north-east India carried out during 2004–2006 (long before ACT was introduced as a first line of drug for treating Pf cases in north-east India) recorded 95.5% ACPR of ACT with mean parasite clearance time of 55:17±14:26 hrs (RMRC unpublished data). It was significant that the study revealed 4.5% overall failure (Late clinical failure 2.1% and Late parasitological failure 2.4%). This might be due to the resistance of circulating Pf strain to SP, since most of the treatment failures in ACT is related to the failure of SP [40]. In the same study delayed parasite clearance time of 69:49±6:58 hrs was observed at one of study sites indicating relatively slower effectiveness of ACT in that area. Though delayed parasite clearance time is regarded as the most important surrogate signal for artemisinin resistance [41] in the absence of pharmaco-kinetic study, it is difficult to pin point whether increased parasite clearance time observed in that study was due to the resistance to artesunate or SP. However, more recently (in 2013) the National Vector Borne Disease Control Programme, Government of India has changed its drug policy to ACT-AL co-formulated tablet of artemether - Lumefantrine combination for three days regimen for the treatment of Pf cases of north-east India only [42] whereas in the rest of the country a status - quo of using ACT (artesunate and SP) for three days as before was maintained.

A very high level of resistance to CQ and growing resistance to SP, as evident in the present study through therapeutic evaluation and molecular characterization of circulating Pf strains, is a serious concern for malaria control in north-east India. In view of increasing resistance to SP, the partner drug in the ACT combination therapy used in north-east India, and considering this region as a gateway for the drug resistant Pf strains from South-east Asia to the South Asia, a close monitoring on the anti-malarial drug resistance status is necessary with a constant and area wide surveillance for the therapeutic efficacy study along with screening of additional molecular markers for artemisinin and multidrug resistance (such as *pfatp*6, *pfmdr*1 D1042N and *pfmdr*1 Y1246D etc.,) for overall assessment of the dynamics of therapeutic failure of anti-malarials and longitudinal resistance monitoring in north-east India to treat Pf malaria cases. Again the availability of the recently introduced new ACT-AL combination drugs and proper implementation of the new drug policy in NE India needs to be monitored from time to time to make the change more effective and to reduce the emergence of drug resistance as inappropriate use of drugs as well as strong suspicion of widespread use of substandard and/or spurious drugs in this region is believed to increase drug selection pressure and thus lead to development of drug resistance.

## Supporting Information

Figure S1 **Epidemiological situation of malaria in NE India (1961–2012) (Source NVBDCP of NE States, India).**
(TIF)

Figure S2 **Trend of SfR & CFR (10^−5^), NE India (1986–2012) (Source NVBDCP of NE States, India).**
(TIF)

Figure S3 **Prevalence of** ***dhfr*** **and** ***dhps*** **codon mutations in NE India (given in box) as found in present study compared to rest of Indian and SE Asian scenario (Curtsey:** http://www.wwarn.org/surveyor **).**
(TIF)
