# Supplementary figures and images for: Molecular Evidence of Increased Resistance to Anti-Folate Drugs in Plasmodium falciparum in North-East India: A Signal for Potential Failure of Artemisinin Plus Sulphadoxine-Pyrimethamine Combination Therapy
Source: PLoS One. 2014 Sep 3;9(9):e105562. doi: 10.1371/journal.pone.0105562 (PMC4153584; doi:10.1371/journal.pone.0105562)

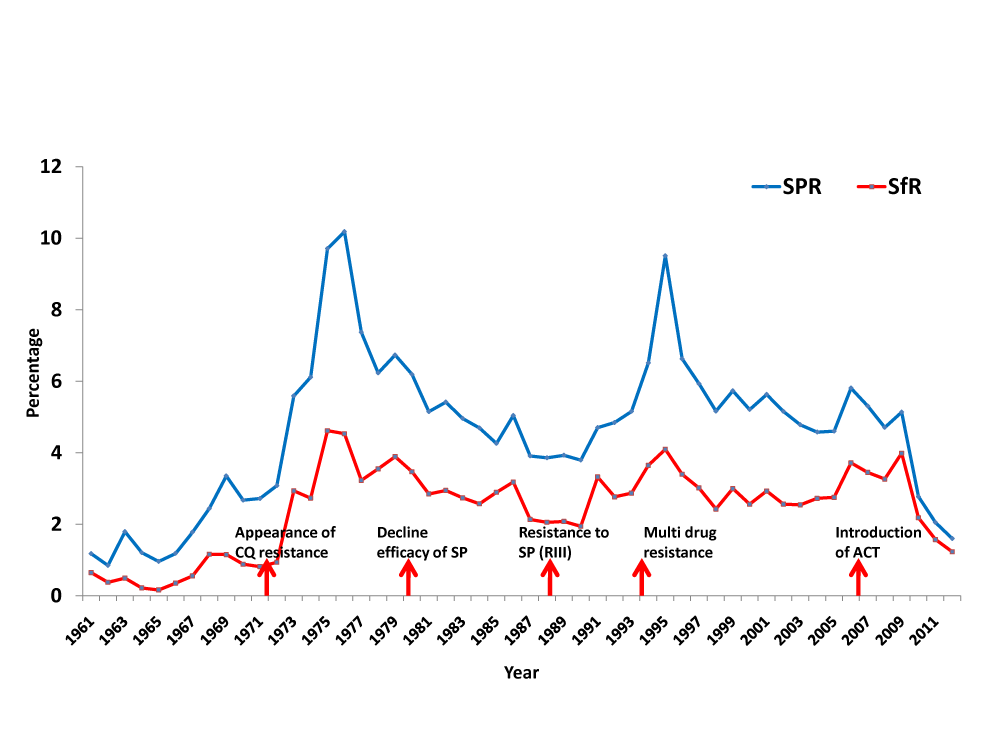

Supplement: Figure S1 — Epidemiological situation of malaria in NE India (1961–2012) (Source NVBDCP of NE States, India). (TIF) [file pone.0105562.s001.tif]

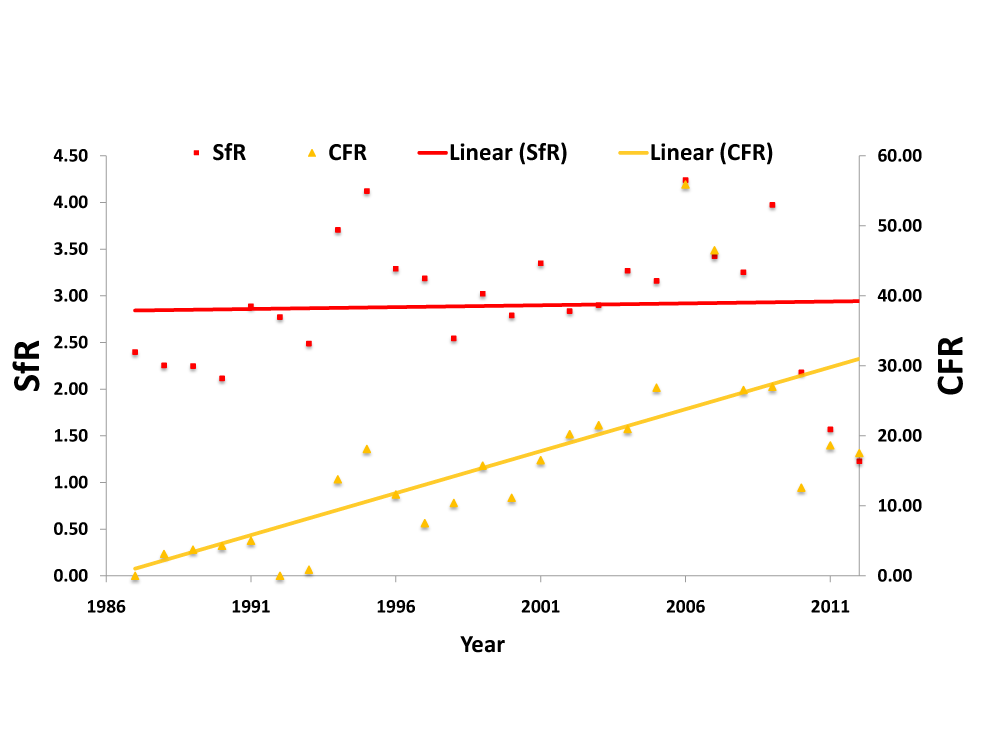

Supplement: Figure S2 — Trend of SfR & CFR (10−5), NE India (1986–2012) (Source NVBDCP of NE States, India). (TIF) [file pone.0105562.s002.tif]

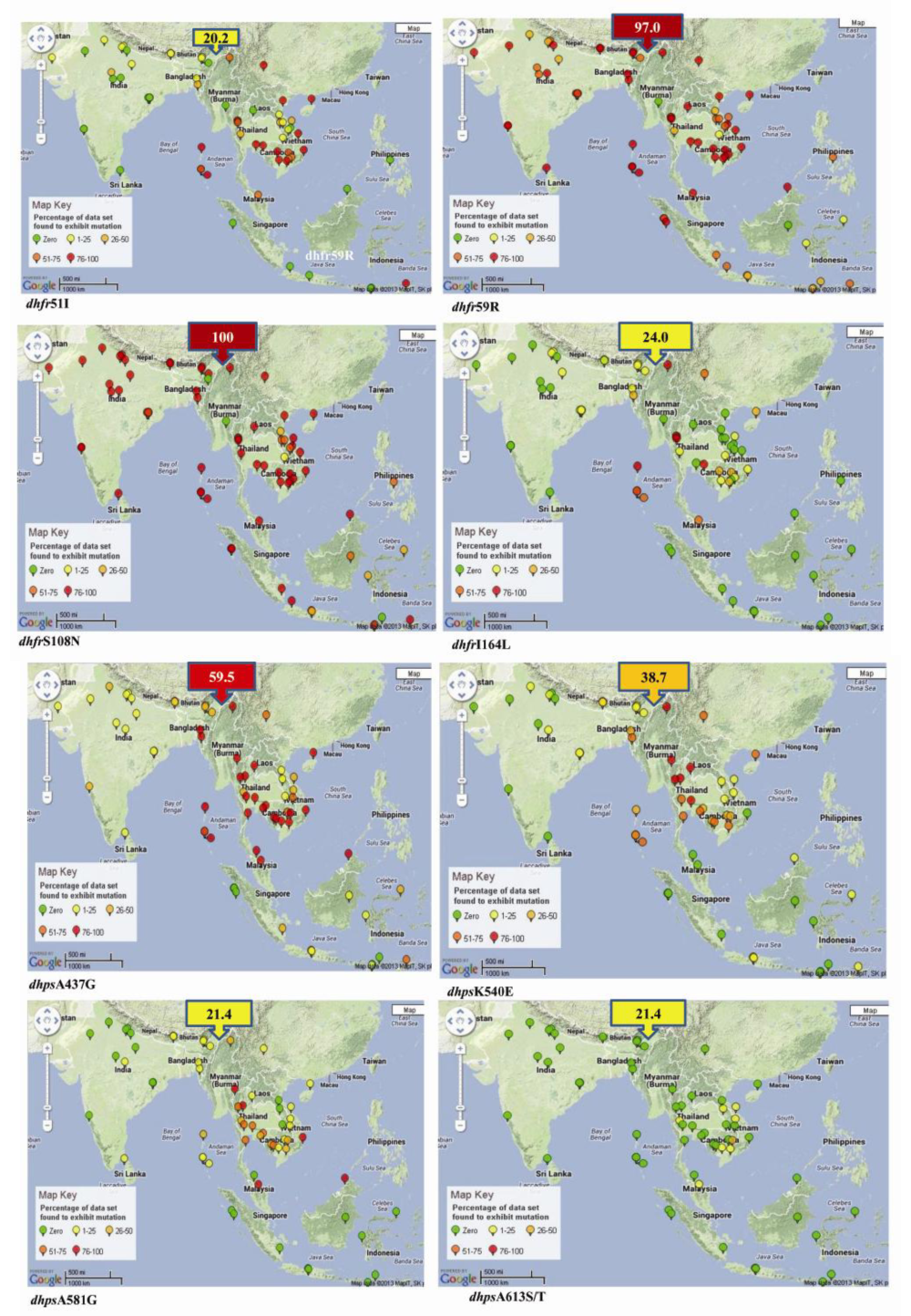

Supplement: Figure S3 — Prevalence of dhfr and dhps codon mutations in NE India (given in box) as found in present study compared to rest of Indian and SE Asian scenario (Curtsey: http://www.wwarn.org/surveyor ). (TIF) [file pone.0105562.s003.tif]
